# Attitudes of Primary School Teachers and Its Associated Factors Toward Students With Attention Deficit Hyperactivity Disorder in Debre Markos and Dejen Towns, Northwest Ethiopia

**DOI:** 10.3389/fped.2022.805440

**Published:** 2022-05-06

**Authors:** Haile Amha, Telake Azale

**Affiliations:** ^1^Department of Nursing, College of Health Science, Debre Markos University, Debre Markos, Ethiopia; ^2^Department of Psychiatry, University of Gondar, Gondar, Ethiopia

**Keywords:** attitudes, attention deficit hyperactivity disorder, associated factors, Ethiopia, primary school, children

## Abstract

**Background:**

Although most instructors appear to understand visible disability, they appear to have a negative attitude toward children with attention deficit hyperactivity disorder (ADHD), considering them to be lazy or purposefully disruptive. In Ethiopia, there is a scarcity of data on teachers’ attitudes toward children with ADHD.

**Methods:**

An institution-based cross-sectional study was conducted. A pre-tested questionnaire that contains a case vignette was administered through face-to-face interview with 417 teachers. The data was entered into Epi-data version 4.2 and exported into SPSS version 25.0 for analysis. Multiple linear regression analyses were used to assess the correlates of attitude in the participants and a B coefficient with 95% confidence interval (CI) were used. The statistical significance was accepted at *p*-value < 0.05.

**Results:**

The mean score of the teachers’ attitude toward ADHD was 41.6 ± 5.4 (95% CI; 41.12, 42.16) and 46% of the participants had unfavorable attitudes. Low level of educational status, knowledge, teaching experience, familiarity in teaching students with ADHD, and having training were significantly associated with attitude of the teachers.

**Conclusion:**

The study revealed that nearly half of the participants had an unfavorable attitude toward students with ADHD. Based on the findings, it was recommended that it is better to strengthen training of teachers to recognize students with ADHD.

## Background

Attention deficit hyperactivity disorder (ADHD) is a neuropsychiatric disorder that affects preschoolers, children, adolescents, and adults worldwide (1). It is characterized by a pattern of decreased sustained attention and increased impulsivity or hyperactivity. General Psychiatrists or other mental health professionals in Ethiopia use Diagnostic Statistical Manual (DSM) criteria to diagnose ADHD (2).

An attitude is a broad, long-term assessment of a person, thing, or issue that can be based on emotive, cognitive, or behavioral information and can vary in strength (3, 4). Attitudinal obstacles are thought to be at the heart of all other environmental barriers, and they are the most difficult to overcome. Misconceptions, preconceptions, labeling, fear of the unknown, resistance, misunderstanding people’s rights and opportunities, and further isolation of children with disabilities are all manifestations of these issues (5).

Teachers in low-income countries, like Nigeria (6), had a more negative attitude toward students with ADHD than teachers in high-income countries, like Germany (7). As a result, teachers’ roles become even more critical in low-income nations, where parents may lack access to information and other resources to help their children with ADHD (6, 8–13). In many countries, one of the most difficult problems for educators is students’ attention and behavior problems. Teachers are important sources of information when it comes to diagnosing ADHD, and 77% of doctors attempted to obtain a written report from the schools of children who had been referred for ADHD treatment (14, 15).

Teachers are responsible for creating learning environments that are responsive to the needs of all the students; additionally, they play pivotal roles in the referral of students for ADHD assessments; however, they report inept skills in managing disruptive classroom behavior and rely on ineffective punishment and warning, removal, suspension, and parent-teacher conferences to do so. However, if teachers have a positive attitude, they will undoubtedly establish a constructive learning atmosphere in which students can engage in difficult conduct while maintaining a reasonable outlook and putting forth effort to achieve learning objectives (16, 17). A person who lacks knowledge may be cautious and seek information, whereas a person who has formed an incorrect opinion may not seek additional information and may offer inappropriate advice. As a result, teachers’ attitudes will influence how they interact with ADHD students, and their negative behavior may have a negative impact on students’ outcomes. In addition, studies have established that teachers’ attitudes toward ADHD have a powerful impact on students’ future achievements, social relationships, and self-esteem (7, 18–20).

A cross-cultural comparative cross-sectional study on 264 German and 264 South Korean teachers using 23 items showed that the mean score of teachers’ attitudes was 57.59 ± 5.16 and 50.70 ± 5.03, respectively, that is 68.56% of German teachers had more favorable attitudes toward students with ADHD as compared to 60.35% Korean teachers (7). Nigerian Survey Study through the sample of (250) primary school teachers (125 men and 125 women) who were randomly selected from across twenty (20) schools by using 20 items, the questionnaire revealed that the mean score teachers’ attitude was 42.26 ± 4.79 (men = 42.62 ± 4.88, women = 41.89 ± 4.68) (9). Factors, such as age, gender, educational level, ethnicity, duration of teaching, knowledge, training, information, and experience of teaching students with ADHD, significantly affected teachers’ attitudes toward ADHD (7, 9, 10, 21–23). According to research, adults who were diagnosed with ADHD as children have reported that a teacher’s caring attitude, extra attention, and guidance were “turning points” in helping them to overcome their childhood problems (24).

There is a scarcity of evidence in Sub-Saharan Africa, particularly in Ethiopia, on teachers’ attitudes toward ADHD. As a result, this study examined teachers’ attitudes and the factors that influence them in primary schools in Debre Markos and Dejen towns.

## Materials and Methods

### Study Setting and Population

This research was carried out in the primary schools of Debre Markos and Dejen Town, both of which are located in the East Gojjam zone. Debre Markos town is located 299 kilometers northwest of Addis Abeba and 255 kilometers east of Bahirdar, the Amhara Region’s capital city. According to 2007 National Census Reports, Debre Markos town has a total population of 62,497 people, of which 32,576 are women and 29,921 are men. Approximately, 97% of the population said they practice Orthodox Christianity. Furthermore, Amhara made up 97.12% of the town’s ethnic composition. There are fourteen primary schools, with a total of 511 teachers and 14,037 students. Dejen is also located in the East Gojjam zone, 65.1 kilometers from Debre Markos, and has a population of 15,483 people, with 7,688 men and 7,795 women, respectively. There are two elementary schools in the town with 224 teachers.

### Sample Size and Sampling Methods

The number of samples required for the study is calculated by using a single population mean formula, using the following assumptions


$$n={\frac{{\left(\frac{Z\,\alpha }{2}\right)}^{2}\,\delta }{d^{2}}}^{2}$$

Where, *n* = minimum sample size, Z_α__/2_ = *Z*-value at (∞ = 0.05) = 1.96, δ^2^ = standard deviation of the mean score of attitude, SD from a previously published study in Nigeria is 4.79 (13), *D* = Margin of error (0.47). After adding 10% of non-respondent, the total sample size for this study was 439. The number of teachers from each school was determined based on their population proportion. Each study subject was selected by using a random sampling technique from the list of teachers in each school. For eligible participants who were not found in the schools, the data collector revisited the schools at reputed time during the data collection period.

### Eligibility Criteria

All teachers who were working at primary schools of Debre Markos and Dejen towns were included and teachers who worked in administrative duties and were not in direct and daily contact with students were excluded from the study.

### Measurements

Attitude toward ADHD was assessed by asking 12 questions of the ADHD belief scale based on a 5-point Likert Scale (with 1 = strongly disagree, 2 = disagree, 3 = neutral, 4 = agree, and 5 as strongly agree) (25). The Cronbach alpha of this tool is 0.84.

The knowledge scale consists of 12 “Yes, No” items, which assess teachers’ knowledge of ADHD and four items in experience-related factors (8). The Cronbach alpha of the tool is 0.78.

These instruments were selected because they use items that have been empirically supported and are agreed-upon measures of general rather than specialized knowledge and attitude of ADHD; in addition, the language used in the instruments and their reasonable number of items to be suitable for the target sample. Teachers with serious illnesses and hearing problems were excluded from the study.

### Data Collection Procedure

Questionnaire preparation was conducted by the authors by adapting from other literature and using standard tools before conducting a pre-test. Data were collected using a pretested, structured and standardized questionnaire through face-to-face interview with five BSc nurses and supervised by two BSc psychiatry nurses. All of the sampled primary school teachers who were present on the day of the survey had been contacted and informed about the study. There was an opportunity to ask questions, and clarifications were provided. The interview process took about 20–30 min to complete. Case vignettes were created by incorporating major symptoms of ADHD that occur in school settings to increase the questionnaires’ understandability and minimize misinterpretation.

### Case Vignette

Student X has been one of your students and you have spent a lot of time with him/her. He/she is often quite fidgety and seldom sits still, even when talking one–on–one with you. He/she abruptly shifts in activity, lack of organization in his work, jumping up in the class, and always talks more than any other students. Often interrupts when you or someone else in a group is talking. He/she is busy doing multiple tasks and appears not to be paying full attention to what you say. He/she is easily distractible, failure to finish tasks, and has poor concentration. He/she seems increasingly irresponsible: coming late for class, not following the instructions. He/she usually forgot what you told and loses important learning materials, and other items. Recently, you were informed that He/she has a diagnosis of ADHD (45).

### Data Quality Assurance

To maintain consistency, the questionnaire was written in English, translated into the local language (Amharic), and then back into English. Prior to data collection, the questionnaire was pretested on 5% of the sample size to ensure its understandability and reliability. Some amendments to the questionnaire and case vignette preparation were done based on the pretest results. Data collectors and supervisors received 1 day of training on the study instrument, data collection procedure, and confidentiality ethical principles. Each day, the supervisors and principal investigator reviewed and checked the collected data for completeness and relevance. The final manuscript was edited by proficient and experienced English language experts.

### Data Processing and Analysis

After checking the collected data for completeness; the data were entered into Epi-data version 4.2 and exported into SPSS version 25.0 for analysis. Descriptive statistics Frequency, Mean, and Standard deviation were used to describe sociodemographic factors and to assess teachers’ attitudes. Basic assumption tests of linear regression (normality, linearity, homoscedastic, and multicollinearity) were done accordingly. Simple and multiple linear regression analyses were used to assess the correlates of attitude in the participants. The *t*-test and analysis of variance (ANOVA) were applied to compare the mean difference of teachers’ attitudes by category of different variables. The coefficients of determination (*R*^2^) and unstandardized adjusted B coefficient with 95% confidence interval were used. Variables with *p*-value less than 0.25 in bivariate analysis were entered into multiple linear regression. The statistical significance was accepted at *p*-value of 0.05.

### Ethical Consideration

Ethical clearance was obtained from the joint Ethical Review Committee (ERC) of the University of Gondar and Amanuel Mental Specialized Hospital, and a Letter of cooperation has been delivered to Debre Markos and Dejen Town educational bureau to get a letter of permission for each school. The study does not incur any cost or expenses on the study participants apart from time cost. There are no potential risks that have caused any harm in any form to the study participants. Anyone who was not willing to participate in the study had not been forced to participate. A letter of cooperation had been given to secure the permission of the schools. After obtaining permission from the school directors, informed (written) consents were obtained from each participant. The study participants were also provided with information about the objectives and expected outcomes of the study. Information obtained from individual participants had been kept secure and confidential.

## Results

### Socio Demographic Characteristics of Respondents

Initially, this study proposed to interview 435 individuals. But data were collected only from 417 teachers making a response rate of 95% in 17 primary schools. The mean age of the respondents was 43.15 ± 9.63 years ranges from 22 to 58 years. More than half of the respondents 251 (60.2%) were women. The mean duration of teaching experience was 20.32 ± 10.23 years ranges from 4 months to 38 years. Nearly three-fourth of women, i.e., 304 (72.9%) were married and 71 (17.0%) were single. The major ethnic group was Amhara 402 (96.4%) followed by Oromo 11 (2.6%). The greater proportion of the respondents was Orthodox by religion 389 (93.3%) followed by Protestant 20 (4.8%). The majority of teachers 373 (89.4%) have a Diploma and 29 (7.0%) attended university education in the Degree level (Table 1).

**TABLE 1:** Sociodemographic characteristics of primary school teachers of Debre Markos and Dejen towns, 2018.

Variables	Categories	Frequency percentage	
Age (Mean ± SD)			43.15 ± 9.63
Duration of teaching	(Mean ± SD)		20.32 ± 10.23
Sex	Male	166	39.8
	Female	251	60.2
Religion	Muslim	8	1.9
	Orthodox	389	93.3
	Protestant	20	4.8
Marital status	Single	71	17.0
	Married	304	73.0
	Separated	6	1.4
	Divorced	18	4.3
	Widowed	18	4.3
Ethnicity	Amhara	402	96.7
	Oromo	11	2.6
	Tigre	3	0.7
Educational level	Diploma	373	89.4
	Bachelor degree	29	7.0
	Certificate	15	3.6

*n = 417.*

### Information and Knowledge About Attention Deficit Hyperactivity Disorder

There were 409 (98.1%) teachers who reported having no ADHD-related training. The number of teachers who reported knowing something about the disorder was 49(11.8%). The source of information for those who reported having information was from multiple sources. A total of 23 (40.4%) teachers got information from friends followed by 15 (26.3%) from TV/radio and 8(14%) got information from two sources. More than half of the teachers 230(55.2%) have poor knowledge toward ADHD (Table 2).

**TABLE 2:** Attention deficit hyperactivity disorder-related knowledge of primary school teachers of Debre Markos and Dejen towns, 2018.

Variables	Categories	Frequency	Percentage
Knowledge	Good	187	44.8
	Poor	230	55.2
Training	Yes	8	1.9
	No	409	98.1
Information	Yes	49	11.8
	No	368	88.2
Source of information	Friends	23	40.4
	TV or Radio	15	26.3
	Workshops	12	21.0
	Books	5	8.8
	Journals	2	3.5

*n = 417.*

### Contact With Attention Deficit Hyperactivity Disorder Students

About 89 (21.3%) had experience of teaching students with ADHD. Among them 41 (46.1%) teach 1–2, 21 (23.6%) teach 6–10, 15 (16.9%) teach 3–5, and 12 (13.5%) teach more than 10 students with ADHD.

### Teachers’ Attitude Toward Attention Deficit Hyperactivity Disorder

The mean score of teachers’ attitude toward ADHD was 41.6 ± 5.4 (95% CI; 41.12, 42.16) as measured by the ADHD belief scale that ranges from 12 to 60. Less than half of the respondents 192 (46%) scored below the mean score (had unfavorable attitude). There is a significant mean difference among participants by their age, educational level, and teaching experience, however, there is no significant mean difference when we compare by their sex (Table 3).

**TABLE 3:** Comparation of mean score of attitudes of primary school teachers by socio-demographic variables in Debre Markos and Dejen towns, 2018.

Variables	Category	*N*	Mean	Std. Deviation	Test	*p*-value
Sex	Male	166	40.88	5.62	*T*-Test	0.681
	Female	251	42.14	5.16		
Educational status of teachers	Diploma	373	41.56	5.00	ANOVA	<0.05
	Degree	29	46.86	5.57		
	Certificate	15	33.60	2.50		
Teaching experience	<5	44	37.09	4.29	ANOVA	<0.05
	5–10	36	36.47	3.57		
	11–20	111	39.03	3.77		
	>20	225	44.66	4.49		
Age	18–25	30	37.17	4.49	ANOVA	<0.05
	26–35	73	36.97	3.72		
	36–45	100	39.15	3.80		
	46–60	214	45.01	4.20		

*n = 417.*

### Factors Associated With Attitude of Teachers Toward Attention Deficit Hyperactivity Disorder

In simple linear regression model variables, such as sex, age, educational level, teaching experience, source of information (information from friends, information from TV/radio, information from books, and no information), knowledge, training, experience of teaching students with ADHD, and number of ADHD students (not teaching students with ADHD, teaching 1–2, teaching 3–5, teaching 6–10, and teaching >10 students with ADHD), had *p-*value below 0.05. The *p*-value < 0.25 is used as a cut point to select candidate variables, which enter to multiple linear regression from a simple one.

In this study, the total value of VIF was less than 4, and the tolerance was close to 1, thus there was no problem with multicollinearity. Certificate level education, teaching experience, knowledge, training, and experience of teaching students ADHD were significantly associated with the attitude of teachers toward students with ADHD in a multiple linear regression model. A significant *F*-test equation was found [*F*_(12,404)_ = 85.94, *p-*value = 0.001)], with an adjusted *R*^2^ = 0.71, 71% of the variation in an attitude was explained by independent variables in the model (Table 4).

**TABLE 4:** Bivariables and multivariable factors of an attitude and their corresponding explained variability among teachers of Debre Markos and Dejen towns, 2018.

Variables	Crude unstandardized Coefficient B (95% *CI*)	Adjusted unstandardized Coefficients B (95% *CI*)
Certificate	−8.34 (−11.00, −5.67)	−4.92 (−6.44, −3.39) ***
Degree	**ref**	**ref**
Knowledge	0.91 (0.77, 1.05)	0.47 (0.37, 0.56) *
Teaching experience in years	0.38 (0.34, 0.41)	0.18(0.15, 0.22) ***
Information from friends	6.14 (3.94, 8.33)	−0.57 (−2.33, 1.19)
Information from TV or Radio	5.36 (2.62, 8.09)	−0.41 (−2.14, 1.32)
Information from books	6.24(1.51,10.96)	0.06(−2.81, 2.92)
No information recoded	−7.12 (−8.74, −5.50)	−1.40 (−2.94, −0.142) *
Information from workshops	**ref**	**ref**
Training	−11.46 (7.84, 15.07)	2.79 (0.34, 5.24) ***
Having no training	**ref**	**ref**
Experience of teaching students with ADHD	−8.80 (7.87, 9.74)	5.03 (3.27, 5.27) ***
Having no experience of teaching students with ADHD	**ref**	**ref**
Gender male	1.26 (0.21, 2.31)	0.46 (−0.12, 1.05)
Female	**ref**	
Number of ADHD students 3 to 5	7.10 (4.31, 9.90)	−0.03 (−1.82, 1.76)
Number of ADHD students 6 to 10	8.15(5.92, 10.39)	0.7 (−0.84, 2.22)
Number of ADHD Students > 10	**ref**	**ref**

*N-B. ref, reference.*

***p-value < 0.001, *p-value < 0.05, CI, confidence interval.

## Discussion

There have been few studies that investigate teachers’ attitudes toward ADHD. Many of these studies purport to investigate attitudes but instead examine teacher knowledge, or they investigate attitudes toward a specific aspect of ADHD, such as medication use, teacher stress, and ADHD stigma (26–28). The study aims and objectives are important for the region and the findings are highly relevant for the child and adolescent psychiatry practice.

In this study, the mean score of teachers’ attitudes toward students with ADHD was 41.65.4 (95% CI 41.12, 42.16), and 192 (46%) of the participants scored below the mean score of attitudes. This result is in line with a cross-sectional study of Egypt (29) where around 55% of the respondents scored above the mean. It is also consistent with a Nigerian study that reported a mean score of 42.26 ± 4.79 (9). The mean score of attitude in the current study was lower than the study conducted in Saudi Arabia with a mean score of (46.6 ± 4.8) (30), which was assessed by a similar tool as a current study and study done in Israel (31), which revealed that 75% of the participants were above the mean score. Possible explanations include increased awareness-raising activities, the availability of comprehensive workshops and practice-based training, and the inclusion of courses on child behavioral disorders in their educational system. These boost their confidence in teaching and supporting students with ADHD, which contributes to an increase in the mean attitude score.

The result of the current study was lower than studies done in Nigeria (10) and Egypt (22) with mean scores of 93.49 ± 8.14 and 50.28 ± 5.54, respectively. The possible explanation might be the tool difference where they used 30 items questionnaires. The mean score of the current study was also lower than the comparative cross-sectional study done on South Korean and German teachers where the mean scores were 50.70 ± 5.03 and 57.59 ± 5.16, respectively (7). The possible reason could be that they used 21 items questionnaires. The finding of the current study was higher than the studies conducted in Jordan (24) and Iran (38) where the mean score of the teacher’s attitude toward ADHD was 5.3 and 18.51, respectively. This could be due to the characteristics of their tool, which were with two options as yes or no, thus it may be resulted with a lower mean score.

Knowledge was a significant predictor of attitudes of teachers implies that study participants who had knowledge resulted in a 0.47-unit increment in attitude compared to those who had poor knowledge (B; 0.47, CI; 0.37, 0.56). This could be because of the greater the extent of teachers’ knowledge, they can appropriately guide their evaluations and behavior, and hence their attitudes become more favorable. This result is supported by studies conducted in Iran (32), South Africa (33), Nigeria (9), and Egypt (22). On the contrary to the current study, research conducted in Jordan (21) revealed that knowledge was not associated with attitude. The possible reasons might be a methodological gap where they used a convenience sampling technique and the dichotomizing tool, which was used to assess the attitude of teachers.

The current study revealed that training was positively associated with attitude of teachers toward students with ADHD. Study participants who had training were associated with an increment of 2.79 units (B; 2.79, CI; 0.33, 5.24) in attitude as compared with those who had no ADHD-related training. The possible reasons could be that training programs provide opportunities for teachers to reflect on their feeling, enable them to develop a critical understanding, and realistic expectations, to adapt the teaching procedure accordingly for students with ADHD, which could alter their negative attitude. This is supported by studies conducted in Germany and South Korea (7), Saudi Arabia (34), Nigeria (10), and Egypt (22).

Teaching experience is positively associated with an attitude such that, adjusting for the other variables in the model, for each additional year in the duration of Teaching, teachers’ attitude was predicted to be increased by 0.18 units (B; 0.18, CI; 0.15, 0.22). The possible explanation could be, through experience teachers have trained themselves in a sense and have developed strategies that they regularly utilize in their classrooms to support students with ADHD, so that this could enable them to shape their misconceptions. This result is supported by studies conducted in Germany, South Korea (7), and Nigeria (9).

Compared to study participants who were not experienced in teaching students with ADHD, those who had experienced were associated with an increment of 4.27 units in attitude (B; 4.27, CI; 3.27, 5.27). The possible reason could be that when teachers became more experienced in teaching of students with ADHD, their ability to seek information, and training about the disorder will increases that could give an opportunity to shape their misunderstanding. The more time they spent with these students, the higher will be their understanding and knowledge toward students with ADHD. This result is supported by Nigerian (10) and Saudi Arabian studies (35). In the current study, Study participants who had a certificate level of education were associated with a reduction of 4.92 units in attitude as compared to teachers who had a degree (B; −4.92, CI; −6.44, −3.39). The possible explanation could be that teachers with less educational qualification often feels less confident, so the less competent the teachers feel about their teaching abilities, the more negative their attitude may be toward teaching students with ADHD. This is supported by studies done in South Africa (33) and Nigeria (9) where the educational qualification was a significant factor. Based on the finding of this study, it was recommended to enhancement of teachers’ knowledge, correct unfavorable attitudes held by teachers about ADHD through availing information during conferences, and by preparing workshops and training.

### Limitations of the Study

The study had some limitations, as the data were gathered through a face-to-face interview and the study was based on attitudes, it could be prone to social desirability bias. The effect of social desirability bias was suggested by the fact that participants’ mean scores were higher than expected despite low training and knowledge. The cross-sectional study design nature made it difficult to show the temporal relationship of attitude and its associated factors. The researchers of this study suggested that other researchers better to conduct follow-up studies and apply additional data collection methods to explore teachers’ perceptions through qualitative data.

## Conclusion

The study revealed that participants have a lower mean score of attitudes toward ADHD compared to other studies. Nearly half of the study participants scored below the mean score attitude or had an unfavorable attitude. Knowledge, duration of teaching, training, and experience in teaching students with ADHD were predictors of favorable attitude whereas a certificate level education was a predictor of unfavorable attitude.

## Data Availability Statement

The original contributions presented in the study are included in the article/Supplementary Material, further inquiries can be directed to the corresponding author.

## Ethics Statement

The studies involving human participants were reviewed and approved by the Ethical clearance was obtained from joint Ethical Review Committee (ERC) of University of Gondar and Amanuel Mental Specialized Hospital and Letter of cooperation has been delivered to Debre Markos and Dejen town educational bureau in order to get a letter of permission for each school. A letter of cooperation had been given to secure the permission of the schools. After obtaining permission from the school directors, and informed (written) consents were obtained from each participant. The study participants were also provided with information about the objectives and expected outcomes of the study. Information obtained from individual participants had been kept secure and confidential. The patients/participants provided their written informed consent to participate in this study.

## Author Contributions

HA conceptualized the proposal, searched the literature, trained the field researchers for data collection, wrote the results and discussion sections, and drafted the first manuscript. TA contributed to the design of the study and provided advice as regards methods, data interpretation, analysis, and also critically reviewed and edited the manuscript. Both authors read and approved the final manuscript.

## Conflict of Interest

The authors declare that the research was conducted in the absence of any commercial or financial relationships that could be construed as a potential conflict of interest.

## Publisher’s Note

All claims expressed in this article are solely those of the authors and do not necessarily represent those of their affiliated organizations, or those of the publisher, the editors and the reviewers. Any product that may be evaluated in this article, or claim that may be made by its manufacturer, is not guaranteed or endorsed by the publisher.

## Abbreviations

ADHD: attention deficit hyperactivity disorder
AMSH: Amanuel Mental Specialized Hospital
CI: confidence interval
FMOE: Federal Minister of Education
FMOH: Federal Minister of Health
MH GAP-IG: mental health gap action programme intervention guide
SASA: scale for ADHD-specific attitude
SD: standard deviation
SPSS: statistical package for social science
SRQ: self-reporting questionnaire
Uog: University of Gondar
US: United States
VIF: variance inflation factor.
