# Supplementary material for: Attitudes of Primary School Teachers and Its Associated Factors Toward Students With Attention Deficit Hyperactivity Disorder in Debre Markos and Dejen Towns, Northwest Ethiopia
Source: Front Pediatr. 2022 May 6;10:805440. doi: 10.3389/fped.2022.805440 (PMC9120593; doi:10.3389/fped.2022.805440)
Supplement: Supplementary file 1 [file Data_Sheet_1.docx]

**Annex-consent form**

Questionnaire on attitude and factors associated with Attention Deficit Hyper Activity

Disorder among primary school teachers of Debre Markos and Dejen towns, Northwest

Ethiopia.

**Dear Participants,**

My name is ; I am hereby in the behalf of Haile Amha who is a student undertaking a Master’s degree in Mental Health given jointly between the University of Gondar and Amanuel mental specialized hospital. One of the requirements for the degree is to conduct a research project. This letter serves to ask consent from you to take part in this research. The purpose of the research is to assess attitude towards attention deficit hyperactivity disorder among primary school teachers. Your participation in this research is voluntary. If you decide not to participate there will be no negative consequences for you. If you do decide to participate there will be no benefits for you. There is no any risk will occur to you because of your participation in this study. All the responses given by you and results obtained will be kept confidential using coding system whereby no one will have access to your response. Without permission from you and legal body any part of this study will not be disclosed to the third person. You have full right to refuse and withdrawal to participate in this study if you don’t wish. The interview period will take about

40 minutes. The participants are selected by chance. If you are willing to participate in this study, you need to understand and sign the agreement form, and then you will be asked to give your responses to data collectors.

Name of investigator: Haile Amha: phone number-0947055139

[Email-haileleul19@gmail.com](mailto:Email-haileleul19@gmail.com)

Name of advisors: 1. Telake Azale (PhD, Associated professor)

Are you voluntary to participate in the interview? Yes No

**Informed consent form**

I hereby confirm that I understand the contents of this document and the nature of the research project, and I consent to participate voluntarily in the research project. I understand that I am at autonomy to withdraw from the project at any time.

Signature of Participant ----------------------------Date------------------------------------------ Name and signature of supervisor ----------------------------------------- Date---------------- Name and signature of data collector --------------------------------------Date----------------

**10.2. The data collection instrument English version**

**INTRODUCTION**: Thank you for agreeing to take part in this study. This study is intended to determine teachers’ knowledge and attitude towards attention deficit hyperactivity disorder. You are not expected to give your name or phone number. Every data obtained from you will be kept confidential. Without Permission from you and legal body any part of this study will be disclosed to the third person.

**INSTRUCTION**: The questionnaire has four parts. It will take about 20-30 minutes to complete the interview. In order to increase understandability of the questionnaires there is a case vignette about Attention Deficit Hyper Activity Disorder initially Please try to respond all questions. Thank you very much for your patience.

**A. Socio demographic information**

| S no | Socio demographic information | Responses |
| --- | --- | --- |
| 101. | How old are you? | years |
| 102 | Gender | 1.Male  2.Female |
| 103 | What is your religion | 1.Orthodox  2.Muslim  3.Protestant  4.other |
| 104 | Marital status | 1. Single  2. Married  3.Separated  4.Divorced  5. Widowed |
| 105 | What is your ethnicity | 1.Amhara  2.Oromo  3.Tigre  4.Others ---------- |
| 106 | What is your Level of Education? | 1.Diploma  2.Bachelor’s degree  3. Postgraduate degree  4.other |
| 107 | How many Years of experience do you have  being a teacher | months  years |

39

**Case Vignette**

Student x has been one of your students and you have spent a lot of time with you. He/she is often quite fidgety and seldom sits still, even when talking one–on–one with you. He/she abruptly shifts in activity, lack of organization in his work, jumping up in class always talks more than any other students. Often interrupts when you or someone else in a group is talking. He/she is busy doing multiple tasks and appears not to be paying full attention to what you say. He/she is easily distractible, failure to finish tasks and has poor concentration He/she seems increasingly irresponsible: coming late for class, not following through on instructions. He/she usually forgot what you told and loses important learning materials and other items. Recently, you were informed that He/she has a

diagnosis of attention deficit hyperactivity disorder.

40

**B. Attitudes of Teachers towards ADHD**

| **S.**  **No** | **Statement** | **Strongly**  **Disagree** | **Disagree** | **Neutral** | **Agree** | **Strongly**  **Agree** |
| --- | --- | --- | --- | --- | --- | --- |
| 1 | Training teachers in behavioral  management is important |  |  |  |  |  |
| 2 | Improving the parents’ skill would benefit  their children |  |  |  |  |  |
| 3 | ADHD child should be treated if  recommended by a doctor |  |  |  |  |  |
| 4 | Social skill training can be helpful for a  child with ADHD |  |  |  |  |  |
| 5 | Behavioral management is an effective  treatment |  |  |  |  |  |
| 6 | Clear consistent rules and consequences  are helpful in treating ADHD |  |  |  |  |  |
| 7 | Teaching techniques are helpful in  managing ADHD |  |  |  |  |  |
| 8 | ADHD can be the result of the child not  trying to control his/her behavior |  |  |  |  |  |
| 9 | ADHD results from parents being  inconsistent with rules and consequences |  |  |  |  |  |
| 10 | Family problems may contribute to a  child’s ADHD |  |  |  |  |  |
| 11 | I want to learn specialized teaching  techniques to treat an ADHD child |  |  |  |  |  |
| 12 | Children develop ADHD as they need  attention |  |  |  |  |  |

41

**C. Teachers’ Knowledge of ADHD**

| s.no | Statement | Responses |
| --- | --- | --- |
| 1 | ADHD children have a biological and genetic predisposition | 1.Ye  2.No |
| 2 | ADHD is not a serious problem and does not need to be managed | 1.Yes  2.No |
| 3 | ADHD can be caused by poor parenting practices and parental spoiling | 1.Yes  2.No |
| 4 | ADHD-related difficulties are lifelong | 1.Yes  2.No |
| 5 | Specially trained educators should teach these ADHD children | 1.Yes  2.No |
| 6 | The same discipline and rules used for all children should be applied to  ADHD  children | 1.Yes  2.No |
| 7 | Educators should be aware of ADHD and ADHD children in the class | 1.Yes  2.No |
| 8 | ADHD students should receive less homework than others | 1.Yes  2.No |
| 9 | ADHD can be treated and managed with proper medication | 1.Yes  2.No |
| 10  11 | ADHD can often be caused by sugar or food additives  Chaotic and dysfunctional family is the etiology of ADHD | 1.Yes  2.No  1.Yes  2.No |
| 12 | Being able to watch television or play with computers for minutes or hours  rules  out ADHD diagnosis | 1.Yes  2.No |

42

**D. Experience related factors**

| S no | Experience related factors | Responses |
| --- | --- | --- |
| 1. | Do you have attend any training about  ADHD | 1.Yes  2.No |
| 2 | Do you have an Experience in teaching to a  child with ADHD | 1.Yes  2.No |
| 3 | Number of ADHD students you teach | 1.None  2.1-2  3.3–5  4.6–10  5.10 |
| 4 | Source of information | 1.no information  2.televion/radio  3.friends  4.journals  5.workshops  6.books  7.others |

43

**10.3. Amharic version consent form**

በጎንደር ዩኒቨርሲቲ የህክምና እና የጤና ሳይንስ ኮሌጅ ፣የሳይካትሪ ዲፓርትመንት እና በአማኑኤል አእምሮ ስፔሻላይዝድ ሆስፒታል መካከል የተደረገ የጋራ ፕሮግራም

በየመንቀዥቀዥ እና ትኩረት የማጣት/የአእምሮ መረበሽ ህመም ዙሪያ በደብረ ማርቆስ እና ደጀን ከተሞች የአንደኛ ደረጃ መምህራንን አመለካከት እነ ተዛማጅ ጉዳዮችን ለማጥናት የተዘጋጀ መጠይቅ

የተሳታፊው መረጃ ቅጽ እና የተሳትፎ ማረጋገጫ

ውድ ተሳታፉዎች

እኔ አቶ እባላለሁ፡፡ አቶ ሀይሌ አምሀ በጎንደር ዩኒቨርሲቲ እና አማኑኤል የአእምሮ ልዩ ሆስፒታል መካከል በሚሰጠዉ የሁለተኛ ዲግሪ ፕሮግራም የስነአእምሮ ህክምና ትምህርታቸዉን በመከታተል ላይ ይገኛሉ፡፡ይህ ደብዳቤ በዚህ ምርምር ላይ ተሳታፉ እንዲሆኑ ለመጋበዝ ሲሆን የምርምሩ ዓላማ በየመንቀዥቀዥ እና ትኩረት የማጣት/የአእምሮ መረበሽ ህመም ዙሪያ የአንደኛ ደረጃ መምህራንን አመለካከት አና ተሃማጅ ጉዳዮችን መመዘን ነው፡፡

በዚህ ጥናት ላይ የመሳተፍ ውሳኔው የእርስዎ ነው፡፡ አለመሳተፍ ቢፈልጉ የሚያመጣብዎት ምንም አይነት ችግር አይኖርም፡፡

ለመሳተፍም ከወሰኑ ምንም አይነት ጥቅም አይኖርዎትም፡፡ በጥናቱ ላይ በሚሳተፉበት ወቅት በእርስዎ ላይ የሚደርስ ምንም አይነት ጉዳት የለም፡፡ በዚህ ጥናት ላይ የሚሰጡት ማንኛውም አስተያየት እና መልስ በኮድ ስርዓት በሚስጢራዊነት የሚቀመጥ ሲሆን ለማንም ሰው አይሰጥም፡፡ ካለ እርስዎ ፈቃድ እና ህጋዊ መብት ለሶስተኛ ወገን መረጃው አይተላለፍም፡፡ ለእርስዎ ካልመሰለዎት ከዚህ ጥናት ተሳታፉነት ራስዎን የማግለል ሙለ መብት አለዎት፡፡ ቃለመጠይቁ 40 ደቂቃ የሚፈጅ ሲሆን ተሳታፉዎቹ የሚመረጡት በእድል ነው:: በዚህ ጥናት ላይ ለመሳተፍ ከፈለጉ የስምምነት ፊርማዎ በቅፁ ላይ መፈረም ይኖርብዎታል፡፡ ከዚያም ለዳታ ሰብሳቢዎቹ ምላሽዎን እንዲሰጡ ይጠየቃሉ፡፡

የተመራማሪው ስም፡ሀይሌ አምሃ

ስልክ፡0947055139

የአድቫይዘር ስም፡ 1. ዶ/ር ተላከ አዛለ(ፒኢችዲ)

በቃለ መጠይቁ ላይ ለመሳተፍ ፈቃድዎ ነው? አዎ አይደለሁም

የተሳትፍ ማረጋገጫ

የሰነዱን ይዘት የተረዳሁ ስሆን የምርምር ፕሮጀክቱንም አላማ ተረድቻለሁ፡፡ በዚህ ምርመር ፕሮጀክት ላይም ለመሳተፍ ፈቃደኛ ሆኛለሁ፡፡ በማንኛውም ሰዓትም ከጥናቱ ራሴን የማግለል መብት እንዳለኝ አውቃለሁ፡፡

የተሳታፊ ፉርማ

ቀን

የሱፐርቫይዘር ስም እና ፊርማ ቀን የዳታ ሰብሳቢ ስም እና ፊርማ ቀን

መግቢያ፡ በዚህ አጠር ያለ መጠይቅ ላይ ለመሳተፍ በመወሰንዎ አመሰግናለሁ፡፡ይህ ጥናት በተማሪዎች ላይ የሚታየዉን የአእምሮ ጠና መቃወስ በተመለከተ የመምህራንን እዉቀት እና አመለካከት ለመለካት ታስቦ ነዉ፡፡ በእርስዎ የሚሰጥ ማንኛውም መረጃ በሚስጥራዊነት የሚቀመጥ ይሆናል፡፡ ካለ እርስዎ ፌቃድ ወይም የህግ አካል በስተቀር ይህ መረጃ ለሶስተኛ ወገን አይሰጥም፡፡

መመሪያ፡ መጠይቁ አራት ክፌል አለው፡፡ጥያቂዎችነ በደንብ እንዲረዱአቸዉ በማሰብ የተዘጋጀ ስለ የመንቀዥቀዥ ዕና ትኩረት የማጣት/የአምሮ መረበሽ ህመም ምልክቶችነ የሚገልፅ ምሳሊያዊ ማስረጃ ስላለ ይመልከቱት፡፡ቃለመጠይቁን ለመሙላት 20 ደቂቃ የሚፈጅ ሲሆን ሁለንም ጥያቄዎች ለመሙላት ይሞክሩ፡፡ ስለእግስትዎ እናሰመግናለን፡፡

**10.4. Amharic version questionnaires**

ክፌል **1** የማህበራዊ አኗኗር መረጃዎች

| ቁጥር | መጠይቅ | የኮድ ካታጎሪ |
| --- | --- | --- |
| 101 | ፆታ | 1. ወንድ 2.ሴት |
| 102 | እድሚዎ ስንት ነው? | -----------------አመት |
| 103 | ሀይማኖትዎ ምንድን ነው? | 1.ሙስሉም 3. ፕሮቴስታንት  2.ኦርቶዶክስ 4. 4.ሌላ---------- |
| 104 | የጋብቻ ሁኔታዎ | 1.ያላገባ  2.ያገባ  3.የፈታ/የፈታች  4.የሞተበት/የሞተባት |
| 105 | ብሔርዎ ምንድን ነው? | 1.አማራ 2.ኦሮሞ  3.ትግሬ 4.ሌላ |
| 106 | የትምህርት ደረጃ | 1.ዲፕሎማ 3.ማስተርስ ዲግሪ  2.ዲግሪ 4.ሌላ |
| 107 | የስራ ልምድ በመምህርነት | ወራት  አመት |

ምሳሊያዊ ማስረጃ

ተማሪ x ከእርስዎ ተማሪዎች መካከል ሲሆነ ከእርስዎ ጋርም ብዙ ጊዜ አሳልፈል/አሳልፋለች፡፡ እሱ / እሷ ብዙውን ጊዜ በጣም ቁንጥንጥና አንድ ቦታ ላይ ተረጋግቶ ለመቀመጥ ይቸገራል/ትቸገራለች፡፡ በድንገት እንቅስቃሴን ቶሎ ቶሎ መቀያየር ፣ በክፍል ውስጥ ከወንበር ወንበር መዝለል ፤ ከሌሎች ተማሪዎች የበለጠ ማዉራት፤የሚሰጠዉነ ተግባር በተደራጀ መልኩ ለመስራት መቸገር ይስተዋልባቸዋል፡፡ ብዙ ጊዜ እርስዎ ወይም ሌላ ሰው በቡድን በምትነጋገሩበት ጊዜ ማቋረጥ፤ ብዙ ተግባራትን በአንዴ ለማከናወን መሞከር፤ እርስዎ ለሚናገሩት/ለሚያስተምሩት ነገር ሙሉ ትኩረት አለመስጠት፤በቀላል ነገሮች የሃሳብ መሰረቅ፤ትኩረት ማጣት፤ተግባራት ጀምሮ አለመጨረስ፤ወደ ክፍል ዘግይቶ መምጣት እና ትእዛዛቶችን አለማክበር አዘዉትሮ ይታይባችዋል፡፡ የተጠየቁትን ነገሮች ረስተዉ መምጣት እና ጠቃሚ የትምህርት መሳሪያዎችን እና ሌሎች እቃዎችን መጣል ይታየባችዋል፡፡በቅርቡ, እሱ / እሷ ትኩረት የመሰብሰብና ያለመረጋጋት/የአእምሮ መረበሽ ህመም እንዳለባቸዉ ተነግሮዎታል፡፡

ክፍል **2**፡**-**ስለ የመንቀዥቀዥ ዕና ትኩረት የማጣተ**/**የአምሮ መረበሽ ህመም የመምህራንን አመለካከት በተመለከተ

| ቁ ጥ ር | መጠይቅ | በእጅጉ አልስማማም | በተወሰነ ደረጃ አልስማማም | አላዉቅም | በተወሰነ ደረጃ እስማማለሁ | በከፍተኛ ደረጃ |
| --- | --- | --- | --- | --- | --- | --- |
| 1. | የባህሪ ችግር ያለባቸን ተማሪዎች ለመርዳት/ለመያዝ መምራኖች ስልጠና መውሰዳቸው  አስፈላጊ ነው ፡፡ |  |  |  |  |  |
| 2. | የወላጆችን የአስተዳደግ ችሎታን ማሳደግ ለልጆቻቸዉ አስዳደግ ጉልህ ድርሻ አለው፡፡ |  |  |  |  |  |
| 3. | የመንቀዥቀዥ/ትኩረት የማጣት /የአእምሮ መረበሽ ህመም ያለባቸው ልጆች በሀኪም  መታየትና መታከም አለባቸው፡፡ |  |  |  |  |  |
| 4. | የማህበራዊ ኑሮ ጥበብ/ዘዴ ስልጠና የመንቀዥቀዥ/ትኩረት የማጣት /የአእምሮ  መረበሽ ህመም ያለባቸውነ ልጆች ይረዳችዋል፡፡ |  |  |  |  |  |
| 5. | ባህሪ/ፀባይ ተኮር የስነ ልቦና ህክምና የመንቀዥቀዥ/ትኩረት የማጣት /የአእምሮ  መረበሽ ህመም ላለባቸው ልጆች ፍቱን ህክምና ነው፡፡ |  |  |  |  |  |
| 6. | ግልፅ እና ወጥ የሆኑ ህጎችና ድርጊቶች መከተል የመንቀዥቀዥ/ትኩረት የማጣት  /የአእምሮ መረበሽ ህመም ያለባቸውን ልጆች ለማከም/;ለማረም ይረዳሉ፡፡ |  |  |  |  |  |
| 7. | የማስተማር ዘዴዎች/ብልሃቶች የመንቀዥቀዥ/ትኩረት የማጣት /የአእምሮ መረበሽ  ህመም ያለባቸውን ልጆች ባግባቡ ለመያዝ ይረዳሉ፡፡ |  |  |  |  |  |
| 8. | የመንቀዥቀዥ/ትኩረት የማጣት /የአእምሮ መረበሽ ህመም ልጆች ባህሪያቸውን  /ፀባያቸውን ለመቆጣጠር ያለመፈለጋቸው ዉጤት ነው፡፡ |  |  |  |  |  |
| 9. | የመንቀዥቀዥ/ትኩረት የማጣት /የአእምሮ መረበሽ ህመም የወላጆች ቁጥጥር እነ  ክትትል ማነስ ዉጤት ነው፡፡ |  |  |  |  |  |
| 10. | ቤተሰባዊ ችግሮች ለየመንቀዥቀዥ/ትኩረት የማጣት /የአእምሮ መረበሽ ህመም  አስተዋፆአቸው ከፍተኛ ነው፡፡ |  |  |  |  |  |
| 11. | የመንቀዥቀዥ/ትኩረት የማጣት /የአእምሮ መረበሽ ህመም ያለባቸውን ተማሪዎች  ለመርዳት ልዩ የማስተማሪያ ዘዴዎችን መማር እፈልጋለሁ |  |  |  |  |  |
| 12. | የመንቀዥቀዥ/ትኩረት የማጣት /የአእምሮ መረበሽ ህመም ልጆች ትኩረትነ ለማግኘት  ሲሉ ያዳበሩት ችግር ነው፡፡ |  |  |  |  |  |

47

ክፍል **3**፡**-**ስለ የመንቀዥቀዥ ዕና ትኩረት የማጣተ**/**የአምሮ መረበሽ ህመም የመምህራንን እዉቀት በተመለከተ

| 1 | የመንቀዥቀዥ ዕና ትኩረት የማጣተ/የአምሮ መረበሽ ህመም ተጋላጭነት ከቤተሰብ ሊወረስ ይችላል ብለው ያስባሉ | 1.አዎ  2.የለም |
| --- | --- | --- |
| 2 | የመንቀዥቀዥ ዕና ትኩረት የማጣተ/የአምሮ መረበሽ ህመም እንደ ከባድ ችግር የማይታይ ሲሆነ ህክምናም  አያሰፈልገዉም ብለው ያስባሉ | 1.አዎ  2.የለም |
| 3 | ልጆች ላይ የመንቀዥቀዥ ዕና ትኩረት የማጣተ/የአምሮ መረበሽ ህመም ጥሩ ባልሆነ ያስተዳደግ ችግር ምክንያት  ሊመጣ ይችላል | 1.አዎ  2.የለም |
| 4 | የመንቀዥቀዥ ዕና ትኩረት የማጣተ/የአምሮ መረበሽ ህመም ጋር ተያይዘዉ የሚመጡ ችግሮች ሁሌም /በዘላቂነት  ከታማሚው ጋር ይኖራሉ | 1.አዎ  2.የለም |
| 5 | የመንቀዥቀዥ ዕና ትኩረት የማጣተ/የአምሮ መረበሽ ህመም ያለባቸዉ ተማሪዎች ስለችግሩ ልዩ ስልጠና በወሰዱ  መምህራን መማር አለባቸው | 1.አዎ  2.የለም |
| 6 | የመንቀዥቀዥ ዕና ትኩረት የማጣት/የአምሮ መረበሽ ህመም ያለባቸዉ ተማሪዎች ላይ የሚተገበሩ ህግና ደንቦች  ከሌሎች ጤነኛ ተማሪዎች ጋር ተመሳሳይ መሆን አለባቸው | 1.አዎ  2.የለም |
| 7 | መምህራን ስለ የመንቀዥቀዥ ዕና ትኩረት የማጣት/የአምሮ መረበሽ ህመም እና ችግሩ ስላለባቸዉ ተማሪዎች እዉቅና  ሊኖራቸው ይገባል | 1.አዎ  2.የለም |
| 8 | የመንቀዥቀዥ ዕና ትኩረት የማጣተ/የአምሮ መረበሽ ህመም ያለባቸዉ ተማሪዎች ከሌሎች ተማሪዎች ያነሰ የቤት ስራ  መሰጠት አለባቸው | 1.አዎ  2.የለም |
| 9 | የመንቀዥቀዥ ዕና ትኩረት የማጣተ/የአምሮ መረበሽ ህመም መታከም ይችላል | 1.አዎ  2.የለም |
| 10 | ጣፋጭ ምግቦችን አዘዉትሮ መመገብ የመንቀዥቀዥ ዕና ትኩረት የማጣተ/የአምሮ መረበሽ ህመም መንስኤ ነው | 1.አዎ  2.የለም |
| 11 | ምስቅልቅል ያለና ያልተረጋጋ ቤተሰብ ውስጥ መኖር ለየመንቀዥቀዥ ዕና ትኩረት የማጣተ/የአምሮ መረበሽ ህመም  ያጋልጣል | 1.አዎ  2.የለም |
| 12 | ለድቂቃዎች /ለሳአታት ቴሌቭዥን ማየት ወይንም ኮምፒዉተር ላይ መጫወት የመንቀዥቀዥ ዕና ትኩረት  የማጣተ/የአምሮ መረበሽ ህመም ምልክት ነው | 1.አዎ  2.የለም |

48

ክፍል **4**፡**-**ከልምድ ጋር የተያያዙ ነገሮችን በተመለከተ

| ቁጥር | መጠይቅ | የኮድ ካታጎሪ |
| --- | --- | --- |
| 1 | የመንቀዥቀዥ ዕና ትኩረት የማጣተ/የአምሮ መረበሽ ህመምን በተመለከት ስልጠና ወስደው ያውቃሉ | 1.አዎ  2.የለም |
| 2 | የመንቀዥቀዥ ዕና ትኩረት የማጣተ/የአምሮ መረበሽ ህመም ያለበት ተማሪ አስተምረዉ ያዉቃሉ | 1.አዎ  2.የለም |
| 3 | አዎ ከሆነ መልስዎ በቁጥር ስንት ይሆናሉ? | 1.1-2  2.3–5  3..6–10  4.10 |
| 4 | የመንቀዥቀዥ ዕና ትኩረት የማጣተ/የአምሮ መረበሽ ህመምን በተመለከት መረጃ ከየት ያገኛሉ | 1.መረጃአግኝቼ  አላዉቅም  2.ተለቪዥን ወይም ሬዲዎ  3.ከጋደኛ  4.ከጥናቶች  5.ከዉይይት መድረኮች  6.በማንበብ  7.ሌላ |
